# 2-(1,3-Benzothia­zol-2-yl)-6-eth­oxy­phenol

**DOI:** 10.1107/S160053681103114X

**Published:** 2011-08-06

**Authors:** D. Lakshmanan, R. Madhan Raj, R. Selvakumar, M. Bakthadoss, S. Murugavel

**Affiliations:** a Department of Physics, C. Abdul Hakeem College of Engineering & Technology, Melvisharam, Vellore 632 509, India; b Department of Physics, Ranipettai Engineering College, Thenkadapathangal, Walaja 632 513, India; c Department of Organic Chemistry, University of Madras, Maraimalai Campus, Chennai 600 025, India; d Department of Physics, Thanthai Periyar Government Institute of Technology, Vellore 632 002, India

## Abstract

In the title compound, C_15_H_13_NO_2_S, the benzothia­zole unit is essentially planar [maximum deviation = −0.0099 (5) Å for the S atom] and is oriented at a dihedral angle of 4.8 (5)° with respect to the benzene ring. An intra­molecular O—H⋯N hydrogen bond generates an *S*(6) ring motif. The crystal packing is stabilized by C—H⋯π inter­actions.

## Related literature

For background to the applications of benzothia­zoles in the chemical industry, see: Bradshaw *et al.* (2002 ▶); Delmas *et al.* (2002 ▶); Hutchinson *et al.* (2002 ▶). For the pharmacological activity of benzothia­zole derivatives, see: Repiĉ *et al.* (2001 ▶); Schwartz *et al.* (1992 ▶). For related structures, see: Baryala *et al.* (2010 ▶); Zhang *et al.* (2008 ▶). For hydrogen-bond motifs, see: Bernstein *et al.* (1995 ▶).
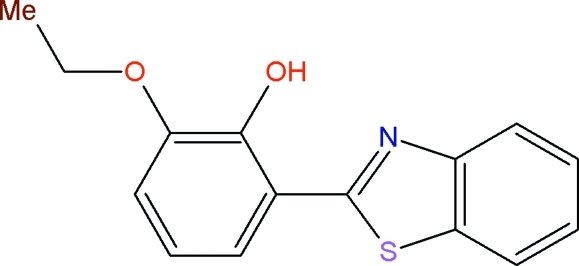


## Experimental

### 

#### Crystal data

C_15_H_13_NO_2_S
*M* *_r_* = 271.32
Monoclinic, 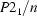

*a* = 9.8739 (5) Å
*b* = 9.6222 (4) Å
*c* = 13.3644 (6) Å
β = 95.269 (2)°
*V* = 1264.37 (10) Å^3^
*Z* = 4
Mo *K*α radiation
μ = 0.25 mm^−1^
*T* = 293 K
0.24 × 0.22 × 0.16 mm

#### Data collection

Bruker APEXII CCD diffractometer
Absorption correction: multi-scan (*SADABS*; Sheldrick, 1996) ▶ *T*_min_ = 0.941, *T*_max_ = 0.960
19076 measured reflections
5191 independent reflections
3449 reflections with *I* > 2σ(*I*)
*R*_int_ = 0.023

#### Refinement

*R*[*F* ^2^ > 2σ(*F* ^2^)] = 0.041
*wR*(*F* ^2^) = 0.125
*S* = 1.01
5191 reflections
173 parameters
H-atom parameters constrained
Δρ_max_ = 0.36 e Å^−3^
Δρ_min_ = −0.21 e Å^−3^

### 

Data collection: *APEX2* (Bruker, 2004 ▶); cell refinement: *APEX2* and *SAINT* (Bruker, 2004 ▶); data reduction: *SAINT* and *XPREP* (Bruker, 2004 ▶); program(s) used to solve structure: *SHELXS97* (Sheldrick, 2008 ▶); program(s) used to refine structure: *SHELXL97* (Sheldrick, 2008 ▶); molecular graphics: *ORTEP-3* (Farrugia (1997 ▶); software used to prepare material for publication: *SHELXL97* and *PLATON* (Spek, 2009 ▶).

## Supplementary Material

Crystal structure: contains datablock(s) global, I. DOI: 10.1107/S160053681103114X/bt5597sup1.cif

Structure factors: contains datablock(s) I. DOI: 10.1107/S160053681103114X/bt5597Isup2.hkl

Supplementary material file. DOI: 10.1107/S160053681103114X/bt5597Isup3.cml

Additional supplementary materials:  crystallographic information; 3D view; checkCIF report

## Figures and Tables

**Table 1:** Hydrogen-bond geometry (Å, °) *Cg*1 and *Cg*2 are the centroids of the C8–C13 and C2–C7 rings, respectively.

*D*—H⋯*A*	*D*—H	H⋯*A*	*D*⋯*A*	*D*—H⋯*A*
O1—H1⋯N1	0.82	1.90	2.626 (1)	147
C14—H14*A*⋯*Cg*1^i^	0.97	2.91	3.779 (1)	149
C14—H14*B*⋯*Cg*2^ii^	0.97	2.65	3.506 (1)	148

Symmetry codes: (i) 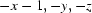; (ii) 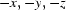.

**Fig. 1.**
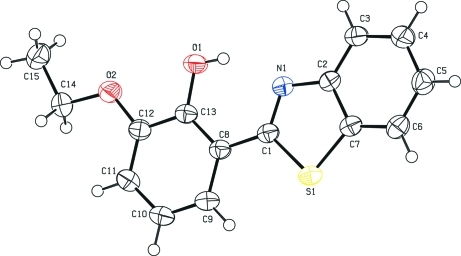
The molecular structure of the title compound with the atom numbering scheme. Displacement ellipsoids are drawn at the 50% probability level. H atoms are presented as a small cycles of arbitrary radius.

**Fig. 2.**
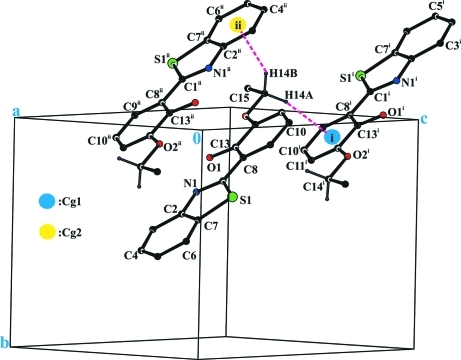
A view of the C-H···π interactions (dotted lines) in the crystal structure of the title compound. Cg1 and Cg2 denotes centroid of the C8-C13 benzene ring and C2-C7 benzene ring, respectively. [Symmetry code: (i) -1-x, -y, -z; (ii) -x, -y, -z.]

## supplementary crystallographic information

### Comment

Benzothiazole are remarkable heterocyclic ring systems. They possess therapeutic value, are synthetic intermediates in the preparation of medicinal compounds and find numerous applications in chemical industry (Bradshaw *et al.,* 2002, Hutchinson *et al.,* 2002, Delmas *et al.,* 2002). Benzothiazole nucleus is associated with several pharmacological activities such as anti-tumor (Repiĉ *et al.,* 2001) and antimicrobial (Schwartz *et al.,* 1992). In view of this biological importance, the crystal structure of the title compound has been determined and the results are presented here.

Fig. 1. shows a displacement ellipsoid plot of (I), with the atom numbering scheme. The benzothiazole moiety (S1/N1/C1-C7) is essentially planar[maximum deviation = -0.0099 (5) Å for the S atom] and lies at an angle 4.8 (5)° with respect to the benzene ring. The geometric parameters of the title molecule agrees well with those reported for similar structures (Baryala *et al.,* 2010, Zhang *et al.,* 2008).

In addition to the van der Waals interaction, the crystal packing is stabilized by O-H···N and C-H···π hydrogen bonds. The intramolecular O1-H1···N1 hydrogen bond generates an S(6) ring motif (Bernstein *et al.,* 1995). The crystal packing (Fig. 2) is stabilized by intermolecular C-H···π interactions, the first one between ethoxy group of atom H14A and the benene ring (C8-C13) (Table 1; C14-H14A···Cg1^i^, Cg1 is the centroid of the C8-C13 ring, symmetry code as in Fig. 2), and the second one between ethoxy group of atom H14B and the benzene ring (C2-C7) (Table 1; C14-H14B..Cg2^ii^, Cg2 is the centroid of the C2-C7 ring, symmetry code as in Fig. 2).

### Experimental

A mixture of 3-ethoxy-2-hydroxybenzaldehyde (0.1g, 0.6 mmol) and 2-aminobenzenethiol (0.075g, 0.6 mmol) was placed in a round bottom flask and melted at 180 ^0^C for 1h. After completion of the reaction as indicated by TLC, the crude product was washed with 5 mL of ethylacetate and hexane mixture (1:49 ratio) which successfully provided the pure product 2-(benzo[d]thiazol-2-yl)-6-ethoxyphenol as colorless solid (91%). The pure compound was crystallized from ethylacetate-hexane 2:10. Single crystals suitable for X-ray diffraction were obtained by slow evaporation of a ethylacetate solution at room temperature.

### Refinement

All the H atoms were positioned geometrically, with O-H = 0.82 Å and and C-H = 0.93 - 0.98 Å and constrained to ride on their parent atom, with *U*_iso_(H)=1.5*U*_eq_ for methyl and hydroxyl H atoms and 1.2*U*_eq_(C) for other H atoms.

### Crystal data

**Table:**

C_15_H_13_NO_2_S	*F*(000) = 568
*M**_r_* = 271.32	*D*_x_ = 1.425 Mg m^−^^3^
Monoclinic, *P*2_1_/*n*	Mo *K*α radiation, λ = 0.71073 Å
Hall symbol: -P 2yn	Cell parameters from 5191 reflections
*a* = 9.8739 (5) Å	θ = 2.5–34.1°
*b* = 9.6222 (4) Å	µ = 0.25 mm^−^^1^
*c* = 13.3644 (6) Å	*T* = 293 K
β = 95.269 (2)°	Block, yellow
*V* = 1264.37 (10) Å^3^	0.24 × 0.22 × 0.16 mm
*Z* = 4	

### Data collection

**Table:**

Bruker APEXII CCD diffractometer	5191 independent reflections
Radiation source: fine-focus sealed tube	3449 reflections with *I* > 2σ(*I*)
graphite	*R*_int_ = 0.023
Detector resolution: 10.0 pixels mm^-1^	θ_max_ = 34.1°, θ_min_ = 2.5°
ω scans	*h* = −15→15
Absorption correction: multi-scan (*SADABS*; Sheldrick, 1996)	*k* = −15→9
*T*_min_ = 0.941, *T*_max_ = 0.960	*l* = −21→19
19076 measured reflections	

### Refinement

**Table:**

Refinement on *F*^2^	Primary atom site location: structure-invariant direct methods
Least-squares matrix: full	Secondary atom site location: difference Fourier map
*R*[*F*^2^ > 2σ(*F*^2^)] = 0.041	Hydrogen site location: inferred from neighbouring sites
*wR*(*F*^2^) = 0.125	H-atom parameters constrained
*S* = 1.01	*w* = 1/[σ^2^(*F*_o_^2^) + (0.0658*P*)^2^ + 0.1388*P*] where *P* = (*F*_o_^2^ + 2*F*_c_^2^)/3
5191 reflections	(Δ/σ)_max_ < 0.001
173 parameters	Δρ_max_ = 0.36 e Å^−^^3^
0 restraints	Δρ_min_ = −0.21 e Å^−^^3^

### Special details

**Table:**

Geometry. All esds (except the esd in the dihedral angle between two l.s. planes) are estimated using the full covariance matrix. The cell esds are taken into account individually in the estimation of esds in distances, angles and torsion angles; correlations between esds in cell parameters are only used when they are defined by crystal symmetry. An approximate (isotropic) treatment of cell esds is used for estimating esds involving l.s. planes.
Refinement. Refinement of F^2^ against ALL reflections. The weighted R-factor wR and goodness of fit S are based on F^2^, conventional R-factors R are based on F, with F set to zero for negative F^2^. The threshold expression of F^2^ > 2sigma(F^2^) is used only for calculating R-factors(gt) etc. and is not relevant to the choice of reflections for refinement. R-factors based on F^2^ are statistically about twice as large as those based on F, and R- factors based on ALL data will be even larger.

### Fractional atomic coordinates and isotropic or equivalent isotropic displacement parameters (Å^2^)

**Table:**

	*x*	*y*	*z*	*U*_iso_*/*U*_eq_	
S1	0.05727 (3)	0.30307 (3)	0.19431 (2)	0.03712 (10)	
N1	0.02751 (10)	0.27133 (10)	0.00112 (7)	0.0326 (2)	
C8	−0.12040 (11)	0.11723 (12)	0.08584 (8)	0.0313 (2)	
C13	−0.18273 (11)	0.06427 (12)	−0.00477 (8)	0.0312 (2)	
O2	−0.33618 (10)	−0.08310 (10)	−0.09512 (6)	0.0439 (2)	
O1	−0.15176 (10)	0.10695 (10)	−0.09587 (6)	0.0424 (2)	
H1	−0.0923	0.1666	−0.0891	0.064*	
C1	−0.01672 (11)	0.22478 (12)	0.08401 (8)	0.0309 (2)	
C12	−0.28288 (12)	−0.03998 (12)	−0.00288 (8)	0.0332 (2)	
C11	−0.31818 (13)	−0.09032 (13)	0.08807 (9)	0.0377 (3)	
H11	−0.3841	−0.1591	0.0893	0.045*	
C2	0.12562 (11)	0.37329 (12)	0.02054 (8)	0.0312 (2)	
C7	0.15529 (12)	0.40571 (12)	0.12266 (8)	0.0328 (2)	
C9	−0.15855 (13)	0.06359 (13)	0.17719 (9)	0.0393 (3)	
H9	−0.1177	0.0977	0.2377	0.047*	
C14	−0.43972 (13)	−0.18786 (12)	−0.09985 (10)	0.0378 (3)	
H14A	−0.5186	−0.1550	−0.0686	0.045*	
H14B	−0.4060	−0.2714	−0.0654	0.045*	
C10	−0.25549 (14)	−0.03849 (14)	0.17758 (9)	0.0408 (3)	
H10	−0.2794	−0.0733	0.2384	0.049*	
C3	0.19474 (13)	0.44135 (13)	−0.05155 (9)	0.0389 (3)	
H3	0.1766	0.4205	−0.1194	0.047*	
C6	0.25186 (13)	0.50573 (13)	0.15417 (9)	0.0400 (3)	
H6	0.2709	0.5271	0.2219	0.048*	
C4	0.29027 (14)	0.53996 (14)	−0.02046 (10)	0.0444 (3)	
H4	0.3370	0.5860	−0.0679	0.053*	
C15	−0.47613 (16)	−0.21685 (16)	−0.20937 (11)	0.0508 (3)	
H15A	−0.5093	−0.1333	−0.2424	0.076*	
H15B	−0.5454	−0.2871	−0.2166	0.076*	
H15C	−0.3970	−0.2487	−0.2392	0.076*	
C5	0.31827 (14)	0.57200 (14)	0.08114 (11)	0.0451 (3)	
H5	0.3831	0.6395	0.1001	0.054*	

### Atomic displacement parameters (Å^2^)

**Table:**

	*U*^11^	*U*^22^	*U*^33^	*U*^12^	*U*^13^	*U*^23^
S1	0.04228 (17)	0.04402 (18)	0.02515 (13)	−0.00088 (12)	0.00359 (11)	−0.00212 (11)
N1	0.0336 (5)	0.0369 (5)	0.0273 (4)	−0.0006 (4)	0.0023 (4)	0.0005 (4)
C8	0.0331 (5)	0.0336 (5)	0.0274 (5)	0.0030 (4)	0.0044 (4)	0.0008 (4)
C13	0.0348 (5)	0.0321 (5)	0.0274 (5)	0.0033 (4)	0.0060 (4)	0.0013 (4)
O2	0.0518 (5)	0.0482 (5)	0.0319 (4)	−0.0162 (4)	0.0042 (4)	−0.0041 (4)
O1	0.0528 (5)	0.0484 (5)	0.0264 (4)	−0.0143 (4)	0.0059 (3)	0.0005 (3)
C1	0.0326 (5)	0.0340 (5)	0.0262 (5)	0.0046 (4)	0.0026 (4)	0.0001 (4)
C12	0.0358 (6)	0.0331 (5)	0.0311 (5)	0.0016 (4)	0.0056 (4)	−0.0016 (4)
C11	0.0400 (6)	0.0372 (6)	0.0371 (6)	−0.0022 (5)	0.0107 (5)	0.0011 (5)
C2	0.0326 (5)	0.0317 (5)	0.0289 (5)	0.0027 (4)	−0.0001 (4)	0.0002 (4)
C7	0.0349 (6)	0.0343 (5)	0.0288 (5)	0.0048 (4)	0.0013 (4)	−0.0011 (4)
C9	0.0448 (7)	0.0467 (7)	0.0267 (5)	−0.0009 (5)	0.0054 (5)	0.0013 (5)
C14	0.0345 (6)	0.0357 (6)	0.0435 (6)	−0.0020 (5)	0.0041 (5)	−0.0015 (5)
C10	0.0466 (7)	0.0464 (7)	0.0308 (5)	−0.0012 (6)	0.0107 (5)	0.0056 (5)
C3	0.0439 (6)	0.0430 (6)	0.0295 (5)	−0.0038 (5)	0.0015 (5)	0.0042 (5)
C6	0.0429 (7)	0.0407 (6)	0.0354 (6)	−0.0005 (5)	−0.0017 (5)	−0.0073 (5)
C4	0.0486 (7)	0.0446 (7)	0.0398 (6)	−0.0084 (6)	0.0028 (5)	0.0065 (5)
C15	0.0511 (8)	0.0542 (8)	0.0453 (7)	−0.0084 (6)	−0.0059 (6)	−0.0035 (6)
C5	0.0467 (7)	0.0403 (6)	0.0473 (7)	−0.0074 (6)	−0.0016 (6)	−0.0038 (5)

### Geometric parameters (Å, °)

**Table:**

S1—C7	1.7318 (12)	C7—C6	1.3925 (17)
S1—C1	1.7539 (11)	C9—C10	1.3718 (18)
N1—C1	1.3067 (14)	C9—H9	0.9300
N1—C2	1.3864 (15)	C14—C15	1.5010 (18)
C8—C13	1.4028 (15)	C14—H14A	0.9700
C8—C9	1.4082 (15)	C14—H14B	0.9700
C8—C1	1.4575 (16)	C10—H10	0.9300
C13—O1	1.3464 (13)	C3—C4	1.3750 (18)
C13—C12	1.4105 (16)	C3—H3	0.9300
O2—C12	1.3600 (14)	C6—C5	1.3809 (19)
O2—C14	1.4330 (14)	C6—H6	0.9300
O1—H1	0.8200	C4—C5	1.3950 (19)
C12—C11	1.3825 (16)	C4—H4	0.9300
C11—C10	1.3881 (18)	C15—H15A	0.9600
C11—H11	0.9300	C15—H15B	0.9600
C2—C3	1.3941 (16)	C15—H15C	0.9600
C2—C7	1.4044 (15)	C5—H5	0.9300
			
C7—S1—C1	89.48 (5)	O2—C14—C15	106.25 (10)
C1—N1—C2	111.47 (9)	O2—C14—H14A	110.5
C13—C8—C9	118.96 (11)	C15—C14—H14A	110.5
C13—C8—C1	119.77 (10)	O2—C14—H14B	110.5
C9—C8—C1	121.26 (10)	C15—C14—H14B	110.5
O1—C13—C8	123.48 (10)	H14A—C14—H14B	108.7
O1—C13—C12	116.80 (10)	C9—C10—C11	120.67 (11)
C8—C13—C12	119.71 (10)	C9—C10—H10	119.7
C12—O2—C14	118.02 (9)	C11—C10—H10	119.7
C13—O1—H1	109.5	C4—C3—C2	118.76 (11)
N1—C1—C8	123.20 (10)	C4—C3—H3	120.6
N1—C1—S1	114.80 (9)	C2—C3—H3	120.6
C8—C1—S1	122.00 (8)	C5—C6—C7	117.51 (11)
O2—C12—C11	125.61 (11)	C5—C6—H6	121.2
O2—C12—C13	114.48 (10)	C7—C6—H6	121.2
C11—C12—C13	119.91 (11)	C3—C4—C5	121.05 (12)
C12—C11—C10	120.22 (11)	C3—C4—H4	119.5
C12—C11—H11	119.9	C5—C4—H4	119.5
C10—C11—H11	119.9	C14—C15—H15A	109.5
N1—C2—C3	125.53 (10)	C14—C15—H15B	109.5
N1—C2—C7	114.76 (10)	H15A—C15—H15B	109.5
C3—C2—C7	119.71 (11)	C14—C15—H15C	109.5
C6—C7—C2	121.56 (11)	H15A—C15—H15C	109.5
C6—C7—S1	128.96 (9)	H15B—C15—H15C	109.5
C2—C7—S1	109.48 (8)	C6—C5—C4	121.42 (12)
C10—C9—C8	120.53 (11)	C6—C5—H5	119.3
C10—C9—H9	119.7	C4—C5—H5	119.3
C8—C9—H9	119.7		
			
C9—C8—C13—O1	−178.87 (11)	C1—N1—C2—C3	178.53 (11)
C1—C8—C13—O1	0.34 (17)	C1—N1—C2—C7	−0.66 (14)
C9—C8—C13—C12	0.68 (17)	N1—C2—C7—C6	179.82 (10)
C1—C8—C13—C12	179.89 (10)	C3—C2—C7—C6	0.58 (17)
C2—N1—C1—C8	179.89 (10)	N1—C2—C7—S1	0.53 (12)
C2—N1—C1—S1	0.49 (13)	C3—C2—C7—S1	−178.70 (9)
C13—C8—C1—N1	−3.48 (17)	C1—S1—C7—C6	−179.43 (12)
C9—C8—C1—N1	175.71 (11)	C1—S1—C7—C2	−0.21 (8)
C13—C8—C1—S1	175.88 (8)	C13—C8—C9—C10	−0.23 (18)
C9—C8—C1—S1	−4.93 (16)	C1—C8—C9—C10	−179.43 (11)
C7—S1—C1—N1	−0.16 (9)	C12—O2—C14—C15	−178.61 (11)
C7—S1—C1—C8	−179.57 (10)	C8—C9—C10—C11	−0.3 (2)
C14—O2—C12—C11	1.21 (18)	C12—C11—C10—C9	0.4 (2)
C14—O2—C12—C13	−179.35 (10)	N1—C2—C3—C4	−179.61 (11)
O1—C13—C12—O2	−0.50 (15)	C7—C2—C3—C4	−0.46 (18)
C8—C13—C12—O2	179.92 (10)	C2—C7—C6—C5	−0.25 (18)
O1—C13—C12—C11	178.97 (11)	S1—C7—C6—C5	178.88 (10)
C8—C13—C12—C11	−0.61 (17)	C2—C3—C4—C5	0.0 (2)
O2—C12—C11—C10	179.49 (12)	C7—C6—C5—C4	−0.2 (2)
C13—C12—C11—C10	0.08 (18)	C3—C4—C5—C6	0.3 (2)

### Hydrogen-bond geometry (Å, °)

**Table:**

Cg1 and Cg2 are the centroids of the C8–C13 and C2–C7 rings, respectively.

**Table:**

*D*—H···*A*	*D*—H	H···*A*	*D*···*A*	*D*—H···*A*
O1—H1···N1	0.82	1.90	2.626 (1)	147
C14—H14A···Cg1^i^	0.97	2.91	3.779 (1)	149
C14—H14B···Cg2^ii^	0.97	2.65	3.506 (1)	148

Symmetry codes:  (i) −*x*−1, −*y*, −*z*; (ii) −*x*, −*y*, −*z*.
